# Supplementary material for: Functional networks of the human bromodomain-containing proteins
Source: Front Bioinform. 2022 Aug 10;2:835892. doi: 10.3389/fbinf.2022.835892 (PMC9580951; doi:10.3389/fbinf.2022.835892)
Supplement: Supplementary file 1 [file Image2.pdf]

Supplementary Figure 2

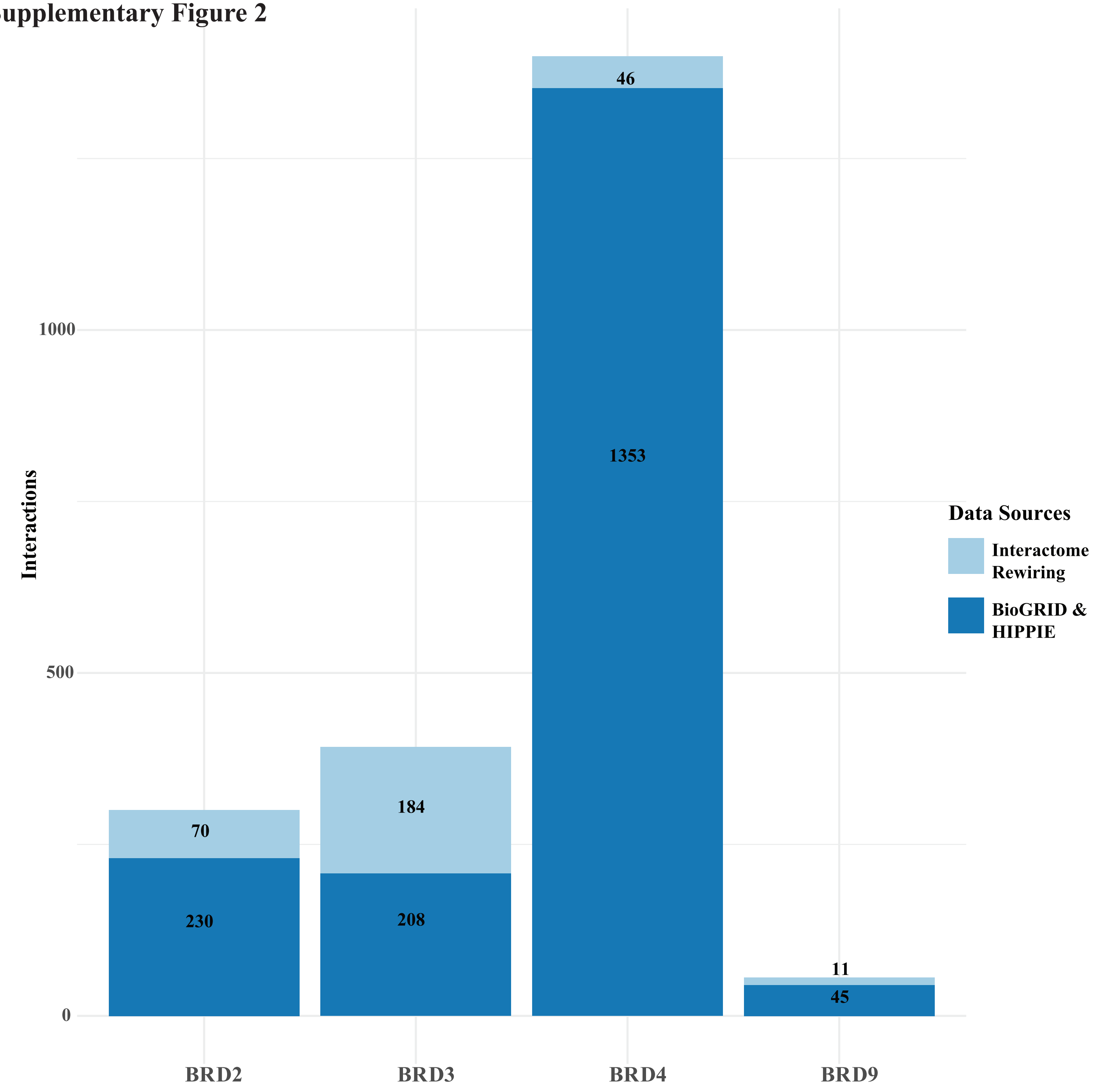

Interaction summary of BRD2,3,4 and BRD9 derived from public databases and the recent publication.
X-axis: 4 different BRD proteins; Y-axis: number of interactions from different sources.
