# Supplementary material for: Functional networks of the human bromodomain-containing proteins
Source: Front Bioinform. 2022 Aug 10;2:835892. doi: 10.3389/fbinf.2022.835892 (PMC9580951; doi:10.3389/fbinf.2022.835892)
Supplement: Supplementary file 2 [file Image3.pdf]

Supplementary Figure 3

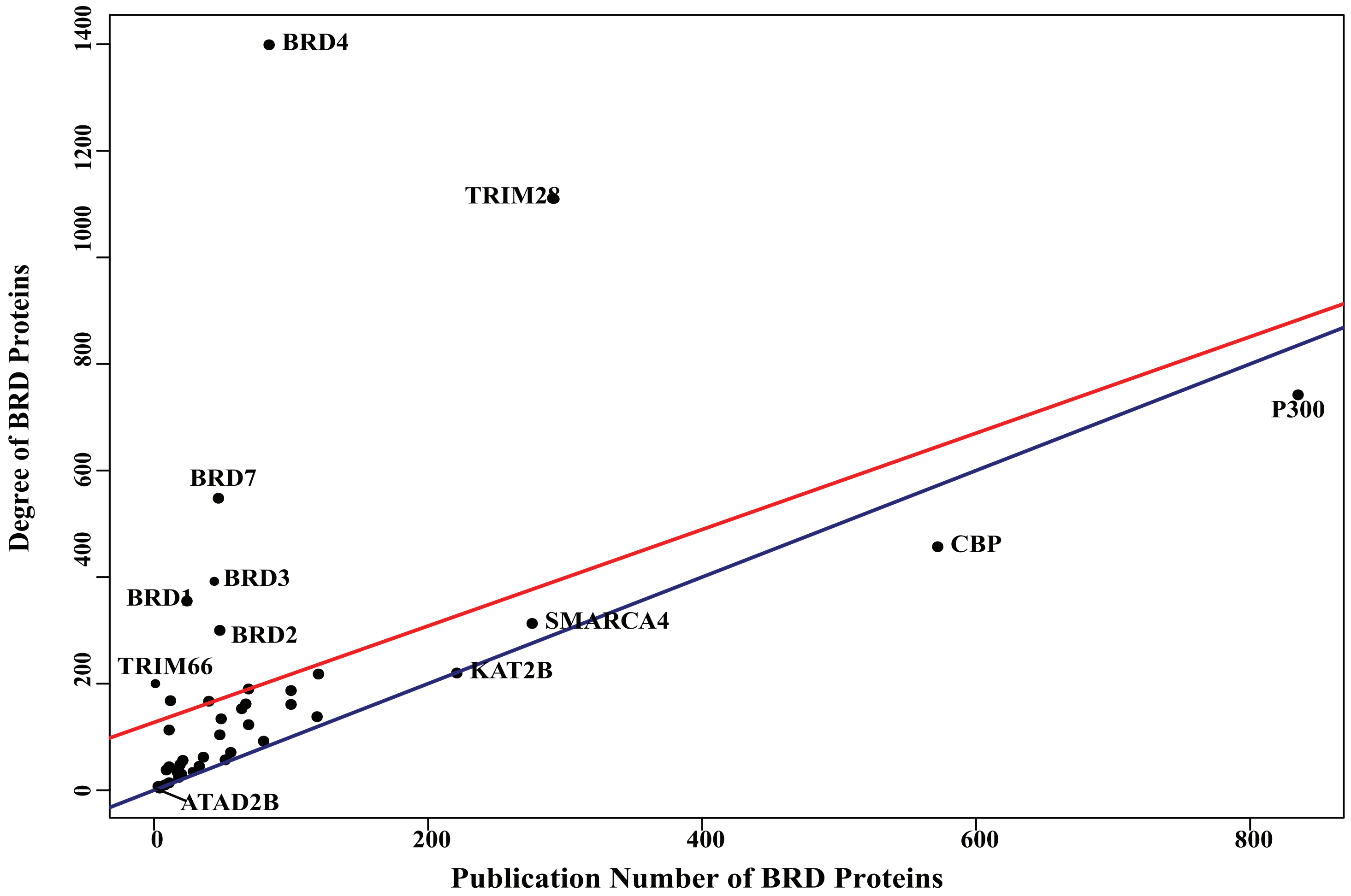

Relationship between degrees and publications for Bromodomain-containing proteins.
Relationship between degrees (y-axis) and publication numbers (x-axis) for BRD proteins, the red line is the linear regression line between degree and publication number and the blue line represents a situation where the degree equals the number of publication (a slope of 1). The top 10 hub BRD proteins are labeled, as well as BRD proteins TRIM66 and ATAD2B.
