# Supplementary material for: Functional networks of the human bromodomain-containing proteins
Source: Front Bioinform. 2022 Aug 10;2:835892. doi: 10.3389/fbinf.2022.835892 (PMC9580951; doi:10.3389/fbinf.2022.835892)
Supplement: Supplementary file 3 [file Table2.docx]

**Supplementary Table 2.**

| Networks | Global PPIN | HuRI Union | BioPlex (HEK 293T) |
| --- | --- | --- | --- |
| Proteins | 19,843 | 9075 | 13,957 |
| Interactions | 559,183 | 63116 | 118162 |
| BRD proteins | 42 | 12 | 31 |
| BRD interactions | 192,785 | 68 | 496 |
