# Supplementary material for: Functional networks of the human bromodomain-containing proteins
Source: Front Bioinform. 2022 Aug 10;2:835892. doi: 10.3389/fbinf.2022.835892 (PMC9580951; doi:10.3389/fbinf.2022.835892)
Supplement: Supplementary file 4 [file Image1.pdf]

Supplementary Figure 1

A

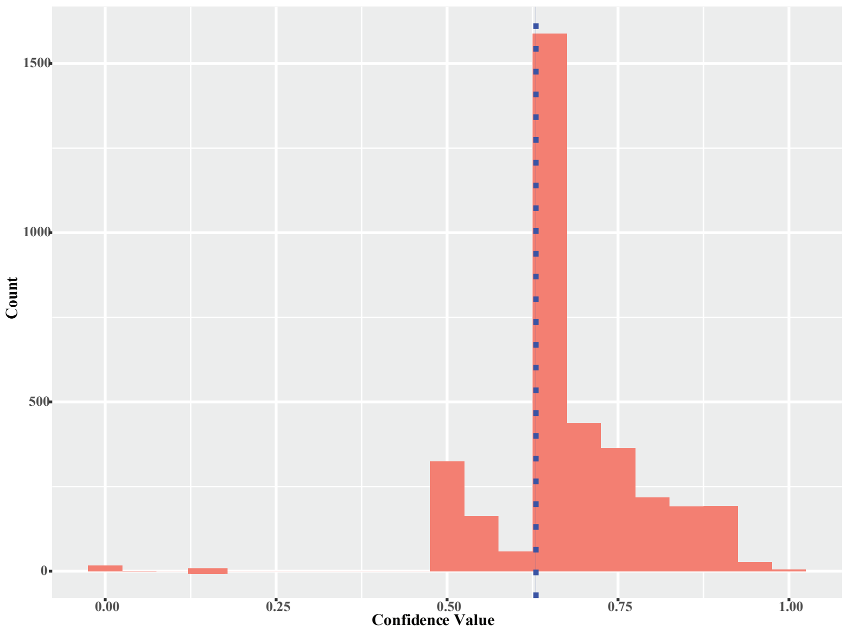

B

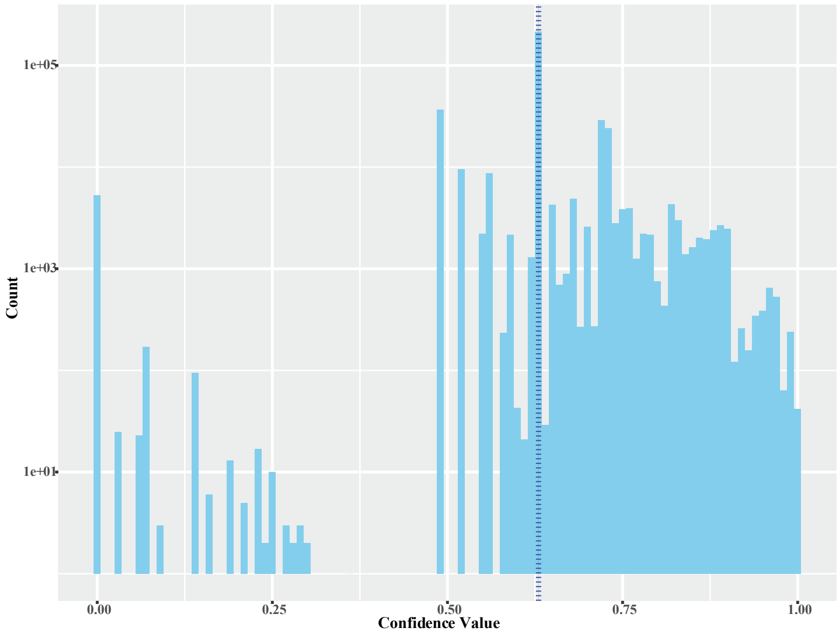

The distribution of confidence value of interactions in HIPPIE.

HIPPIE calculated the confidence value (X-axis) of each interaction based on the publications and techniques used, etc. The blue dotted line refers to the medium confidence value 0.63 suggested by HIPPIE.

A) Confidence value of protein-proteins interactions derived from HIPPIE in the global PPIN.

B) Confidence value of protein-proteins interactions from HIPPIE in BRD PPIN.
